# Supplementary material for: Diversity and functional analysis of rumen and fecal microbial communities associated with dietary changes in crossbreed dairy cattle
Source: PLoS One. 2023 Jan 13;18(1):e0274371. doi: 10.1371/journal.pone.0274371 (PMC9838872; doi:10.1371/journal.pone.0274371)
Supplement: S1 Text — Table A. Roughage-based diet supplemented with dairy meal concentrate. Table B. Chemical composition of the dietary components. Table C. Analysis of Variance (ANOVA) for the 36 metagenomic sequence sample details. Table D. Estimators of diversity within each diet for fecal and ruminal fluid samples (Mean ± Standard Error). Table E. Mean relative abundance (%) of microbial phyla and genus by sample type and diet. (DOC) [file pone.0274371.s001.doc]

# Diversity and functional analysis of rumen and fecal microbial communities associated with dietary changes in crossbreed dairy cattle

Felix M. Kibegwa^1*^, Rawlynce C. Bett^1^, Charles K. Gachuiri^1^, Eunice Machuka^2^, Francesca Stomeo^2#a^, Fidalis D. Mujibi^3^

Table A. Roughage based diet supplemented with dairy meal concentrate

| **Diet** |  | **Rhodes grass Hay** | | **Napier Grass** | | **Kikuyu Grass** | | **Maize Stover** | | **Dairy Meal** | | **Urea** | |  |
| --- | --- | --- | --- | --- | --- | --- | --- | --- | --- | --- | --- | --- | --- | --- |
| Diet 1  (90% roughage and 10% concentrate) | Dry Matter (kg/day) | | 6.97 | | 1.91 | | 0.46 | | 2.32 | | 1.3 | | 0.12 | |
|  | As-Fed (kg/day) | | 7.62 | | 11.48 | | 2.54 | | 2.51 | | 1.47 | | 0.12 | |
| Diet 2  (75% roughage and 25% concentrate) | Dry Matter (kg/day) | | 5.8 | | 1.58 | | 0.39 | | 1.93 | | 3.27 | | 0.12 | |
|  | As-Fed (kg/day) | | 6.35 | | 9.52 | | 2.13 | | 2.08 | | 3.71 | | 0.12 | |
| Diet 3  (60% roughage and 40% concentrate) | Dry Matter (kg/day) | | 4.59 | | 1.25 | | 0.31 | | 1.53 | | 5.2 | | 0.12 | |
|  | As-Fed (kg/day) | | 5.03 | | 7.54 | | 1.68 | | 1.65 | | 5.9 | | 0.12 | |

*Table B. Chemical composition of the dietary components*

|  | **Rhodes grass hay** | **Napier Grass** | **Kikuyu Grass** | **Maize Stover** | **Dairy Meal** | **Urea** |
| --- | --- | --- | --- | --- | --- | --- |
| Dry matter (% As-Fed) | 91.4 | 16.6 | 18.3 | 92.7 | 88.2 | 100 |
| Ash (% DM) | 13.05 | 7.5 | 8.5 | 7.6 | 8.3 | 0 |
| Crude protein (% DM) | 8.32 | 7.3 | 20.3 | 4.8 | 18.4 | 281 |
| Ether extract (% DM) | 3.3 | 3.1 | 3.2 | 1.6 | 4.9 | 0 |
| Crude fiber (CF) | 34.2 | 36.3 | 28.5 | 28.7 | 12.6 |  |
| Nitrogen free extract (NFE) | 41.13 | 45.8 | 39.5 | 57.3 | 55.8 |  |
| Neutral Detergent Fiber (% DM) | 66.25 | 70.2 | 58 | 73 | 0 | 0 |
| Acid Detergent Fiber (% DM) | 40.51 | 43.9 | 36.4 | 49.4 | 0 | 0 |

*Table C. Analysis of Variance (ANOVA) for the 36 metagenomic sequence sample details*

|  | | **Initial Sequences Count** | **Percentage Sequence reduction after QC** | **Percentage of QC Passed Reads with predicted coding sequences** | **Percentage of QC Passed Reads with predicted as rRNA** |
| --- | --- | --- | --- | --- | --- |
| **Fecal** | **Diet 1** | 675381.7 ± 85804.8 | 28.06 ± 1.02 | 73.56 ± 6.15 | 0.53 ± 0.01 |
|  | **Diet 2** | 367796.3 ± 21787.7 | 27.71 ± 1.01 | 81.22 ± 1.93 | 0.56 ± 0.02 |
|  | **Diet 3** | 559739.3 ± 75398.8 | 26.84 ± 1.47 | 80.22 ± 2.83 | 0.44 ± 0.02 |
| **Rumen Liquor** | **Diet 1** | 422497.3 ± 49011.2 | 24.7 ± 1.17 | 78.32 ± 1.87 | 0.4 ± 0.02 |
|  | **Diet 2** | 774926 ± 88819.6 | 25.47 ± 0.79 | 74.6 ± 1.33 | 0.31 ± 0.03 |
|  | **Diet 3** | 786452.7 ± 38767.7 | 28.36 ± 0.73 | 74.54 ± 4.83 | 0 |
| **P – Value** | **S** | 0.023 | 0.127 | 0.401 | 0.002 |
|  | **D** | 0.143 | 0.477 | 0.853 | 0.109 |
|  | **S*D** | 0 | 0.07 | 0.232 | 0.53 ± 0.01 |
| *QC = Quality control, S = Sample Type, D = Diet, S*D = Sample Type x Diet Interaction* | | | | | |

**Table D.** Estimators of diversity within each diet for fecal and ruminal fluid samples (Mean ± Standard Error).

|  | | **Chao-1** | **Simpson (1-D)** | **Shannon (H)** | **Evenness (e^H/S)** | **Equitability (J)** |
| --- | --- | --- | --- | --- | --- | --- |
| **Fecal**^1^ | **Diet 1** | 1713 ± 69 | 0.993 ± 0 | 5.78 ± 0.026 | 0.191 ± 0.009 | 0.777 ± 0.005 |
|  | **Diet 2** | 1675 ± 16 | 0.993 ± 0 | 5.829 ± 0.012 | 0.203 ± 0.003 | 0.785 ± 0.002 |
|  | **Diet 3** | 1729 ± 16 | 0.993 ± 0 | 5.819 ± 0.028 | 0.195 ± 0.005 | 0.781 ± 0.003 |
|  | **p value** | 0.254 | 0.284 | 0.313 | 0.396 | 0.317 |
| **Rumen Liquor**^1^ | **Diet 1** | 1833 ± 9 | 0.954 ± 0.037 | 4.876 ± 0.349 | 0.101 ± 0.017 | 0.622 ± 0.046 |
|  | **Diet 2** | 1786 ± 18 | 0.963 ± 0.002 | 5.22 ± 0.036 | 0.104 ± 0.004 | 0.697 ± 0.005 |
|  | **Diet 3** | 1756 ± 36 | 0.974 ± 0.002 | 5.213 ± 0.069 | 0.105 ± 0.006 | 0.698 ± 0.008 |
|  | **p value** | 0.004 | 0.001 | 0.0003 | 0.23 | 0.246 |
| **P value** | **S** | 0.004 | 0.001 | 4.12E-07 | 3.79E-15 | 1.38E-07 |
|  | **D** | 0.444 | 0.115 | 0.088 | 0.059 | 0.069 |
|  | **SXD** | 0.333 | 0.122 | 0.168 | 0.28 | 0.14 |
| *S = Sample type, D = Diet, SXD Sample type by Diet interaction* | | | | | | |
| *^1^Means are based on 6 cows.* | | | | | | |

**Table E:** Mean relative abundance (%) of microbial phyla and genus by sample type and diet

| **Sample Type** | | **Fecal**^1^ | | | |  | **Rumen Liquor**^1^ | | | |  | **Sample Type**  **P value** |
| --- | --- | --- | --- | --- | --- | --- | --- | --- | --- | --- | --- | --- |
| **Domain** | **Phylum/**Genus | **Diet 1** | **Diet 2** | **Diet 3** | **P value** |  | **Diet 1** | **Diet 2** | **Diet 3** | **P value** |  |  |
| ***Archaea*** | ***Crenarchaeota*** | 0.03 | 0.04 | 0.03 | 0.08 |  | 0.03 | 0.02 | 0.02 | 0.01 |  | NS |
|  | ***Euryarchaeota*** | 1.13 | 1.15 | 0.86 | 0.06 |  | 0.79 | 0.46 | 0.37 | <0.01 |  | * |
|  | *Methanobrevibacter* | 0.31 | 0.19 | 0.13 | <0.01 |  | 0.16 | 0.07 | 0.06 | <0.01 |  | NS |
|  | *Methanosarcina* | 0.16 | 0.18 | 0.13 | 0.02 |  | 0.14 | 0.09 | 0.07 | <0.01 |  | * |
| ***Bacteria*** | ***Firmicutes*** | 52.79 | 45.35 | 46.4 | 0.01 |  | 22.02 | 14.53 | 12.78 | 0.01 |  | ** |
|  | *Clostridium* | 16.07 | 13.91 | 13.05 | 0.04 |  | 5.14 | 3.41 | 2.9 | 0.01 |  | * |
|  | *Eubacterium* | 4.28 | 3.64 | 3.27 | 0.01 |  | 2.02 | 1.25 | 1.09 | <0.01 |  | NS |
|  | *Ethanoligenens* | 1.37 | 1.15 | 1.08 | 0.03 |  | 0.24 | 0.15 | 0.12 | <0.01 |  | ** |
|  | *Faecalibacterium* | 1.05 | 0.94 | 0.9 | 0.2 |  | 0.32 | 0.22 | 0.2 | 0.01 |  | ** |
|  | *Ruminococcus* | 4.47 | 3.86 | 3.52 | 0.01 |  | 2.32 | 1.31 | 1.08 | <0.01 |  | NS |
|  | ***Bacteroidetes*** | 30.61 | 37.63 | 33.91 | 0.06 |  | 61.16 | 41.06 | 32.97 | <0.01 |  | NS |
|  | *Bacteroides* | 16.32 | 18.86 | 17.91 | 0.19 |  | 17.81 | 12.1 | 8.8 | <0.01 |  | NS |
|  | *Prevotella* | 4.03 | 4.93 | 4.36 | 0.09 |  | 32.94 | 21.7 | 16.76 | <0.01 |  | * |
|  | ***Proteobacteria*** | 5.57 | 6.32 | 10.84 | 0.01 |  | 6.97 | 37.53 | 48.63 | <0.01 |  | NS |
|  | *Acinetobacter* | 0.05 | 0.05 | 2.19 | 0.1 |  | 0.33 | 0.36 | 18.78 | <0.01 |  | NS |
|  | *Pseudomonas* | 0.13 | 0.43 | 0.46 | 0.18 |  | 0.32 | 29.02 | 6.27 | 0.01 |  | NS |
|  | ***Actinobacteria*** | 2.69 | 2.46 | 2.43 | 0.02 |  | 1.82 | 1.29 | 1.31 | <0.01 |  | ** |
|  | *Bifidobacterium* | 0.35 | 0.31 | 0.29 | 0.13 |  | 0.27 | 0.16 | 0.14 | <0.01 |  | NS |
|  | *Slackia* | 0.46 | 0.41 | 0.39 | 0.17 |  | 0.29 | 0.18 | 0.16 | <0.01 |  | NS |
|  | ***Spirochaetes*** | 1.13 | 0.86 | 0.72 | 0.08 |  | 0.75 | 0.59 | 0.45 | 0.04 |  | NS |
|  | *Treponema* | 0.62 | 0.37 | 0.31 | 0.11 |  | 0.35 | 0.29 | 0.22 | 0.09 |  | NS |
|  | ***Fusobacteria*** | 0.91 | 0.84 | 0.72 | <0.01 |  | 0.48 | 0.32 | 0.25 | <0.01 |  | * |
|  | ***Lentisphaerae*** | 0.48 | 0.71 | 0.27 | 0.07 |  | 0.25 | 0.2 | 0.14 | 0.03 |  | NS |
|  | *Victivallis* | 0.44 | 0.59 | 0.24 | 0.07 |  | 0.22 | 0.18 | 0.13 | 0.03 |  | NS |
|  | ***Cyanobacteria*** | 0.47 | 0.48 | 0.4 | 0.01 |  | 0.48 | 0.36 | 0.29 | 0.01 |  | NS |
|  | ***Fibrobacteres*** | 0.3 | 0.28 | 0.2 | 0.01 |  | 1.63 | 0.59 | 0.36 | <0.01 |  | NS |
| ***Eukaryota*** | **unclassified (derived from *Eukaryota*)** | 0.21 | 0.21 | 0.14 | <0.01 |  | 0.26 | 0.12 | 0.07 | <0.01 |  | NS |
|  | ***Ascomycota*** | 0.12 | 0.1 | 0.09 | <0.01 |  | 0.12 | 0.08 | 0.06 | 0.01 |  | NS |
|  | ***Streptophyta*** | 0.08 | 0.07 | 0.06 | 0.03 |  | 0.07 | 0.05 | 0.04 | 0.03 |  | NS |
|  | ***Chordata*** | 0.05 | 0.06 | 0.05 | 0.24 |  | 0.07 | 0.08 | 0.09 | 0.15 |  | * |
|  | ***Arthropoda*** | 0.01 | 0.01 | 0.02 | 0.46 |  | 0.04 | 0.03 | 0.02 | 0.02 |  | NS |
| ***Viruses*** | **unclassified (derived from *Viruses*)** | 0.2 | 0.13 | 0.16 | 0.04 |  | 0.16 | 0.62 | 0.56 | 0.01 |  | NS |
| *NS = Not Significant, * = Significant at 0.05, ** = Significant at 0.01*  *^1^Means are based on 6 cows.* | | | | | | | | | | |  |  |
