# Supplementary material for: Diversity and functional analysis of rumen and fecal microbial communities associated with dietary changes in crossbreed dairy cattle
Source: PLoS One. 2023 Jan 13;18(1):e0274371. doi: 10.1371/journal.pone.0274371 (PMC9838872; doi:10.1371/journal.pone.0274371)
Supplement: S1 Fig — a. Fecal microbial communities PCOA, and b. Rumen liquor microbial communities. (DOCX) [file pone.0274371.s002.docx]

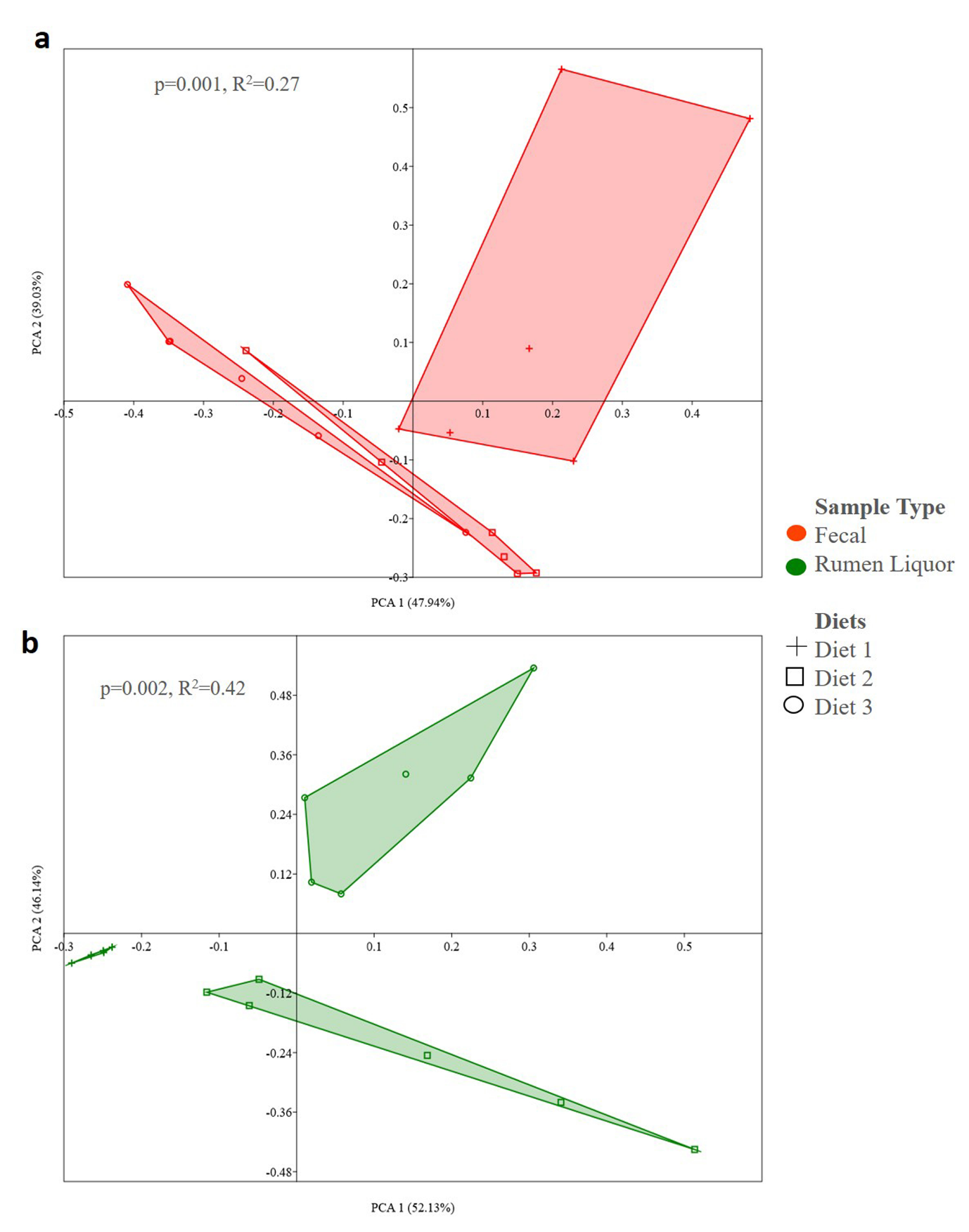


**S2 Fig: Principal component analysis (PCA) of dietary effects on microbial communities within fecal and rumen liquor sample types. a**. Fecal microbial communities PCA, and **b**. Rumen liquor microbial communities
